# Predictive value of dry eye disease signs and corneal in vivo confocal microscopy on serological activity in primary Sjögren disease

**DOI:** 10.1097/MD.0000000000042054

**Published:** 2025-04-04

**Authors:** Yiren Wang, Xiaodan Jiang, Jiaxi Li, Xuemin Li

**Affiliations:** a Department of Ophthalmology, Peking University Third Hospital, Beijing, P.R. China; b Beijing Key Laboratory of Restoration of Damaged Ocular Nerve, Department of Ophthalmology, Peking University Third Hospital, Beijing, P.R. China.

**Keywords:** corneal, dry eye disease, primary Sjögren disease

## Abstract

The purpose of this study is to investigate the differences in the ocular signs and in vivo confocal microscope (IVCM) findings between dry eye disease (DED) patients with and without primary Sjögren disease (SjD), and to establish a predictive model to evaluate the serological activity of SjD based on IVCM findings: we examined 42 (84 eyes) and 41 (82 eyes) with and without SjD, respectively. IVCM was used to evaluate corneal nerve density (CND) and corneal dendritic cell density (DCD). Tear meniscus height (TMH), and tear film breakup time (BUT), corneal fluorescein staining (FL), serum anti-Ro/anti circular single stranded DNA antibody, anti-La/anti single chain binding protein antibody, IgG, C3, and salivary gland biopsies were performed. Patients with SjD had a lower CND (*P* < .0001), lower TMH (*P* < .001), higher DCD (*P* < .001), and higher corneal FL scores (*P* = .014) than patients without SjD. The equation for the prediction of SjD was as follows: Y1 = 4.313 + (−0.23) × CND + 0.02 × DCD + −10.89 × TMH (*P* < .0001). The negative and positive predictive powers of the model were 83.78% and 80.56%, respectively, and the cutoff value for Y1 was 0.392. The predicted serological activity of SjD was as follows: Y2 = −1.271 + 0.014 × DCD + 0.6074 × FL (*P* = .0002). The negative and positive predictive powers of the model were 68.75% and 84.38%, respectively. The cutoff value of Y2 was 0.427. CND, DCD, and TMH demonstrated good predictive values for distinguishing whether DED is caused by SjD. DED and FL had good predictive values for evaluating the serological activity of SjD.

## 1. Introduction

Dry eye disease (DED) is 1 of the most common ophthalmic diseases worldwide. The etiology of DED is diverse, and primary Sjögren disease (SjD) is a relatively special type. DED symptoms and ocular damage are more severe in patients with Sjögren disease than in those with other types of DEDs, and previous studies have shown that inflammatory reactions play an important role in the pathogenesis of DED.^[1,2]^ SJD is a chronic autoimmune disease that can affect tissues and organs, and is characterized by lymphocyte infiltration. Previous studies have suggested that autoimmune antibodies against SjD target the salivary and lacrimal glands^[3]^; additionally, autoimmune antibodies may attack the cornea.

Our previous study^[4]^ demonstrated that corneal dendritic cell density (DCD) was significantly higher in patients with than without SjD. Previous studies^[5]^ have also focused on the correlation between serological autoimmune antibodies and salivary gland biopsy results in patients with SjD and signs of DED. However, how to identify patients with DED caused by SjD, and how to evaluate the activity of SjD remains unknown.

Our study first explored the correlation between the serological markers of SjD and signs of DED; we then performed binary logistic regression analysis to establish a model to predict SjD–DED and serological activity. We aimed to explore the predictive and suggestive effects of the signs of DED on the serological activity of SjD, which is of great significance for identifying patients with DED caused by SjD, as well as formulating appropriate treatment plans.

## 2. Methods

### 2.1. Subjects

This study included 83 patients with DED: 42 with and 41 without SjD. All patients met the following diagnostic criteria for DED: occurrence of any of the following 10 symptoms for at least 3 months: dry eye, burning, foreign body sensation, photophobia, soreness, visual fatigue, tears in the wind, blurred vision, itching, and increased secretion; tear break-up time ≤ 5 s, or tear break-up time between 5 and 10 seconds with fluorescein staining (FL) (+).

The exclusion criteria are as follows: patients with ocular surface diseases such as allergic conjunctivitis or infectious conjunctivitis; history of ocular surgery; age < 18 years; those who have started any form of DED treatment, including eye drops or punctal occlusion.

All DED patients who met the inclusion criteria and did not meet the exclusion criteria were included in this study. For non-SjD–DED patients, no further distinction was made regarding the cause, so non-SjD–DED included both aqueous-deficient and evaporative DED.

The 2016 ACR/EULAR criteria^[3]^ were used to diagnose SjD, and were as follows: anti-anti circular single stranded DNA antibody (SSA)/Ro antibody positivity and focal lymphocytic sialadenitis with a focus score of ≥1 foci/4 mm^2^ (each scoring 3); and an abnormal ocular staining score of ≥ 5 (or van Bijsterveld score of ≥4), a Schirmer test result of ≤ 5 mm/5 minutes, and an unstimulated salivary flow rate of ≤0.1 mL/minute (each scoring 1). Individuals with signs and/or symptoms suggestive of SjD and a total score ≥ 4 for the above items met the criteria for SjD.

The study was performed following the declaration of Helsinki and approved by Peking University Third Hospital’s ethical committee. All participants provided written informed consents to participate and to publish before the study. Participants’ personal information was well protected.

### 2.2. In vivo confocal microscope (IVCM) assessment

The central corneas of all participants were imaged using the Heidelberg retina tomograph 3 in sequence mode (HRT II-Rostock Cornea Module; Heidelberg Engineering Inc., Heidelberg, Germany) under local anesthesia. A drop of carbomer mixed with 0.4% oxybuprocaine hydrochloride was used as a lubricating gel between the eye surface and disposable sterile polymethylmethacrylate cap. The cap was then manually pushed until the gel contacted the previously described central corneal surface. A professional technician obtained at least 100 images of the corneal subbasal layer for each eye, and 3 high-quality images of the central cornea were selected for corneal nerve density (CND) and DCD analyses. An image processing software (Image J; National Institutes of Health, Bethesda) was used to evaluate the corneal nerve and DCD in the IVCM images. NeuronJ and Cell Count tools were used to count the corneal nerves and DCD, and the average corneal nerve and DCD counts of 3 images were recorded.

### 2.3. Other ophthalmic examination

A placido ring-based noncontact corneal topographer (Keratograph 5M; OCULUS, Wetzlar, Germany) was used to evaluate the tear film, including the tear meniscus height (TMH) and tear film breakup time (BUT).^[6]^ Corneal staining scores were assessed by FL using a slit-lamp cobalt blue filter 1 minute after a drop of 2% sterile fluorescein was instilled into the conjunctival sac. Punctate epithelial erosion (PEE) stained with fluorescein was counted and scored; in the absence of PEE, a score of 0 was recorded. If 1 to 5 PEEs were observed, the corneal score was 1; 6 to 30 PEEs received a score of 2, and >30 PEEs received a score of 3. An additional point was added if: PEE occurred in the central 4 mm diameter portion of the cornea; 1 or more filaments were seen anywhere on the cornea; or 1 or more patches of confluent staining, including linear stains, were found anywhere on the cornea. The total FL score of the cornea (PEE grade plus any extra points for modifiers) was noted in the central square of the SICCA ocular staining score form; the maximum possible score for each cornea was 6.^[7]^

### 2.4. Serological markers and salivary gland biopsy

Articaine hydrochloride or 2% lidocaine was used for local infiltration anesthesia in the lower lip surgical area. The patient’s lower lip was fixed, and the surgical site was exposed. Locations with insufficient blood vessels on the medial side of the lower lip were selected, and a horizontal incision or spindle shaped incision of 0.5 to 1.0 cm was made on the mucosa. The incision penetrated the epithelium, and the initial incision of the lip mucosa did not exceed the epithelial layer. The gland was passively peeled off from the surrounding fascia, and the glandular tissue was removed from the surgical area using iris scissors. The tissues were fixed in formalin and examined. At least 4 small salivary gland samples were taken; if the salivary glands were too small (<2 mm), 6 samples with a minimum surface area of 8 mm^2^ were taken. The incision was sutured with 2 to 3 needles and locally sterilized, and the incision was compressed.

Blood sampling was performed to determine the serum anti-Ro/SSA, anti-La/anti single chain binding protein antibody (SSB), IgG, and C3 complement levels.

### 2.5. Statistical analysis

All statistical analyses were performed using SSJD version 26.0 (IBM Corp., Armonk). Continuous variables were described as mean ± standard deviation, or median with interquartile range. Categorical variables were expressed as frequencies and percentages. Continuous variables among the 3 groups were compared using analysis of variance or Kruskal–Wallis tests. Spearman rank order correlation was performed to identify serological markers related to signs of DED, and multiple linear regression analysis was used to evaluate the validity of serological markers and salivary gland biopsies in patients with SjD–DED.

## 3. Results

### 3.1. Clinical data

The demographic characteristics of the patients are presented in Table 1. There were no differences regarding sex (*P* = .106) or age (*P* = .271) between the SjD–DED and non-SjD–DED groups. Salivary gland biopsy and serological anti-SSA and anti-SSB antibodies were negative in patients without SjD.

**Table 1:** Demographic data of patients.

	SjD–DED group	Non-SjD–DED group	*P*-value
Sex			
Male (n, %)	3 (7.14%)	8 (19.05%)	>.05
Female (n, %)	39 (92.86%)	34 (80.95%)	
Age (yr)	49.74 ± 16.02	47.10 ± 14.71	>.05
Diabetes (n, %)	12 (28.56%)	11 (26.19%)	>.05
Hypertension (n, %)	9 (21.42%)	7 (16.67%)	>.05
Salivary gland biopsy (n, %)	22 (52.4%)	0	<.001
Anti-SSA (n, %)	34 (81.0%)	0	<.001
Anti-SSB (n, %)	17 (40.5%)	0	<.001
FL	1.53 ± 1.79	0.89 ± 1.736	<.05
TMH (mm)	0.17 ± 0.05	0.20 ± 0.07	<.05
BUT	4.5 ± 2.9	5.3 ± 2.9	<.05

BUT = breakup time, DED = dry eye disease, FL = corneal fluorescein staining score, SjD = primary Sjögren disease, SSA = anti circular single stranded DNA antibody, SSB = anti single chain binding protein antibody, TMH = tear meniscus height.

### 3.2. Correlation between IVCM results, SjD hematological tests, and salivary gland biopsies

**Figure 1.**
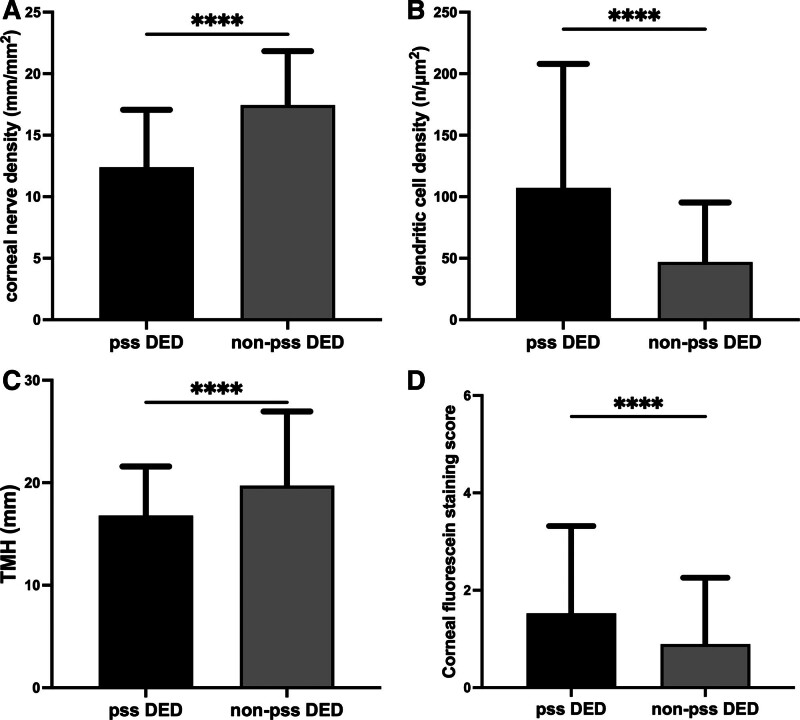
DED signs between the SjD and non-SjD–DED groups. DED = dry eye disease, SjD = primary Sjögren disease.

Correlation analysis was performed between signs of DED, SjD hematological tests, and salivary gland biopsies. Pearson r and *P*-values are shown in Figure 1 and Table 2. CND significantly correlated with anti-SSA (*r* = −.244, *P* = .049) and IgG (*r* = .268, *P* = .027) levels, while DCD significantly correlated with IgG (*r* = .290, *P* = .018) and C3 (*r* = −.297, *P* = .016) levels. TMH significantly correlated with the biopsy results (*r* = .373, *P* = .005), and the FL score significantly correlated with anti-SSA (*r* = .281, *P* = .040) and IgG (*r* = .364, *P* = .007) levels.

**Table 2:** Pearson *r* and *P*-values of the correlation analysis.

	CND		DCD		BUT		TMH		FL	
	*r*	*P*	*r*	*P*	*r*	*P*	*r*	*P*	*r*	*P*
Anti-SSA	−.244	.049^**^	.154	.223	−.013	.915	.155	.199	.281	.040^**^
Anti-SSB	−.103	.710	.081	.523	.053	.667	−.106	.383	.097	.483
IgG	.268	.027^**^	.290	.018^**^	−.171	.159	−.031	.796	.364	.007^***^
C3	−.185	.130	−.297	.016^**^	−.029	.813	.111	.353	−.176	.202
Biopsy	.088	.527	.143	.304	.185	.185	−.373	.005^***^	.197	.236

BUT = breakup time, CND = corneal nerve density, DCD = dendritic cell density, FL = fluorescein staining score, SSA = anti circular single stranded DNA antibody, SSB = anti single chain binding protein antibody, TMH = tear meniscus height.

** *P* < .01.

*** *P* < .001.

### 3.3. Difference in DED signs between patients with and without SjD, and binary logistic regression analysis

The CND (12.39 ± 4.68 mm/mm^2^) and DCD (107.2 ± 100.7 cells/mm^2^) were significantly higher in the SjD–DED than non-SjD–DED group (17.45 ± 4.38 mm/mm^2^ and 46.95 ± 48.36 cells/mm^2^, respectively, *P* < .0001), while the TMH and FL scores were significantly lower in the SjD–DED than non-SjD–DED group (*t* = 3.733, *P* < .001 and *t* = 2.481, *P* = .014, respectively); there was no difference in BUT between the 2 groups (*t* = 0.948, *P* = .345; Table 3 and Fig. 1).

**Table 3:** DED signs in patients with and without SjD.

	SjD–DED group	Non-SjD–DED group	*P*-value
CND (mm/mm^2^)	2.39 ± 4.68	17.45 ± 4.38	<.0001
DCD (cells/mm^2^)	107.2 ± 100.7	46.95 ± 48.36	<.0001
BUT (s)	4.839 ± 2.732	5.265 ± 2.857	.345
TMH (mm)	0.168 ± 0.048	0.202 ± 0.066	<.001
FL	1.565 ± 1.807	0.895 ± 1.362	.014

BUT = breakup time, CND = corneal nerve density, DCD = dendritic cell density, DED = dry eye disease, FL = fluorescein staining score, SjD = primary Sjögren disease, TMH = tear meniscus height.

**Figure 2.**
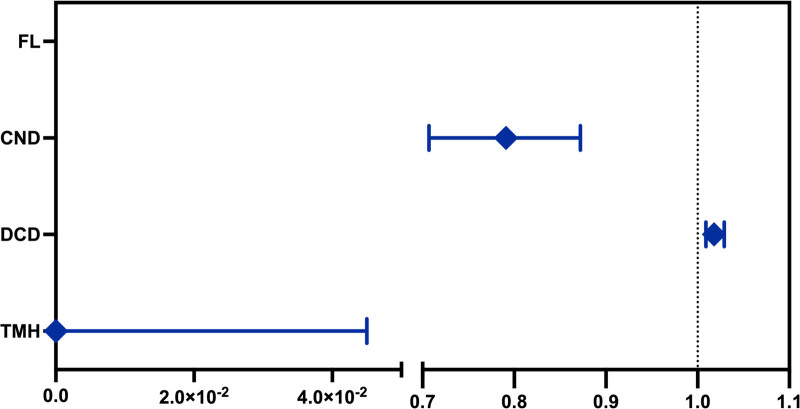
Odds ratio of CND, DCD, and TMH for SjD prediction. CND = corneal nerve density, DCD = dendritic cell density, SjD = primary Sjögren disease, TMH, tear meniscus height.

**Figure 3.**
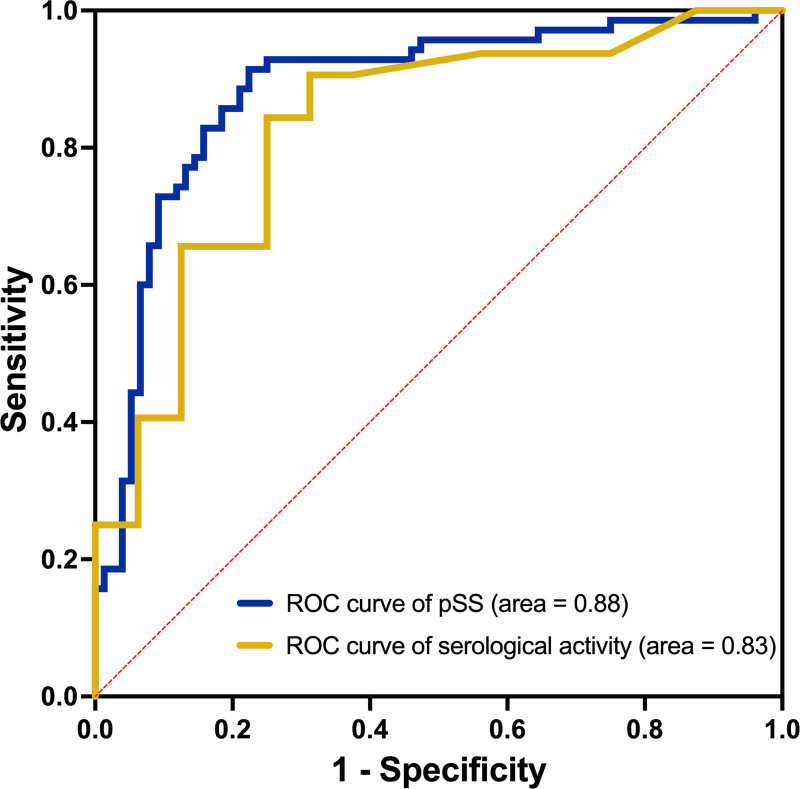
ROC curve of SjD and serological activity prediction. ROC = receiver operating characteristic, SjD = primary Sjögren disease.

Binary regression was conducted on signs of DED and SjD; the results are presented in Table 4 and Figure 2. The *P*-value of the corneal FL score was .73; therefore, it was excluded from the regression equation. The regression equation was as follows: *Y* = 4.313 + (−0.23) *CND + 0.02 × DCD + −10.89*TMH (*P* < .0001). The negative and positive predictive powers of the model were 83.78% and 80.56%, respectively, and the cutoff value for *Y* was 0.392 (sensitivity = 0.914, specificity = 0.777). The receiver operating characteristic (ROC) curve is shown in Figure 3, with an area under the curve (AUC) of 0.884.

**Table 4:** Binary logistic regression of DED signs and SjD.

	Exp (B)	OR	95% CI of OR	*P*-value
CND	−0.23	0.791	0.7067 to 0.8717	<.0001
DCD	0.02	1.018	1.009 to 1.029	<.0001
TMH	−10.89	1.856e−005	2.068e−009 to 0.04521	.0005
Intercept	4.313	74.63	9.501 to 750.8	.0092

CND = corneal nerve density, DCD = dendritic cell density, DED = dry eye disease, OR = odds ratio, SjD = primary Sjögren disease, TMH = tear meniscus height.

### 3.4. Difference in DED signs between serologically active and inactive patients, and binary logistic regression analysis

**Figure 4.**
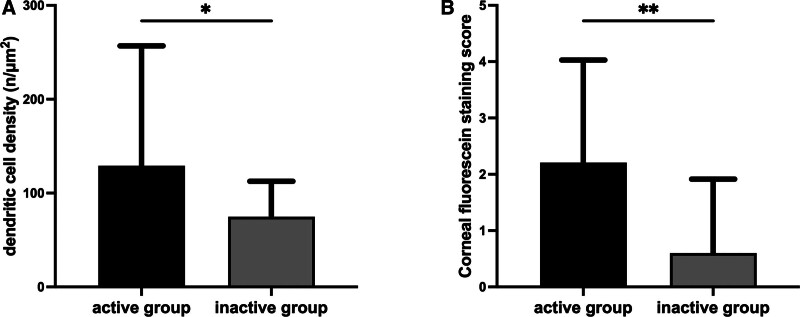
DCD and FL in serologically active and inactive patients. DCD = dendritic cell density, FL = fluorescein staining score.

According to the biologic domain of ESSDAI scoring, patients with SjD–DED were divided into a serologically active (hypocomplementemia, IgG < 5 g/L, or IgG > 16 g/L) and inactive (both complement and IgG are normal) groups. As shown in Table 5 and Figure 4, the DCD was significantly higher in the serologically active than inactive group (129 ± 127.74 cells/mm^2^, and 74.76 ± 37.79 cells/mm^2^, respectively; *P* = .015); the corneal FL score was also significantly higher in the active than inactive group (2.21 ± 1.82, and 0.60 ± 1.31, respectively; *P* < .001).

**Table 5:** DED signs among patients in the serologically active and inactive groups.

	Serologically active group	Serologically inactive group	*P*-value
CND (mm/mm^2^)	11.10 ± 3.51	12.50 ± 3.96	.144
DCD (cells/mm^2^)	129.11 ± 127.74	74.76 ± 37.79	.015
BUT (s)	4.54 ± 2.32	5.30 ± 2.98	.243
TMH (mm)	0.16 ± 0.03	0.18 ± 0.06	.052
FL	2.21 ± 1.82	0.60 ± 1.31	<.001

BUT = breakup time, CND = corneal nerve density, DCD = dendritic cell density, DED = dry eye disease, FL = fluorescein staining score, TMH = tear meniscus height.

**Figure 5.**
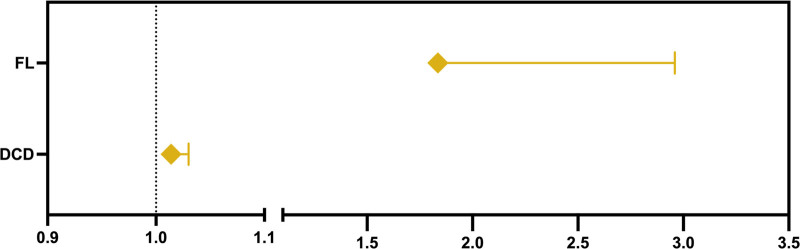
Odds ratio of DCD and FL for SjD serological activity. DCD = dendritic cell density, FL = fluorescein staining score, SjD = primary Sjögren disease.

Binary logistic regression was performed between the signs of DED and SjD serological activity. The results are presented in Table 6 and Figure 5. The regression equation was *Y* = −1.271 + 0.014 × DCD + 0.6074 × FL (*P* = .0002). The negative and positive predictive powers of the model were 68.75% and 84.38%, respectively, and the cutoff value of *Y* was 0.427 (sensitivity = 0.907, specificity = 0.688). The ROC curve is shown in Figure 3, with an AUC of 0.827.

**Table 6:** Binary logistic regression of DED signs and SjD.

	Exp (B)	OR	95% CI of OR	*P*-value
DCD	0.02	1.01	1.00 to 1.03	<.001
FL	0.61	1.84	1.15 to 3.40	<.001
Intercept	−1.27	0.28	0.06 to 1.04	.082

DCD = dendritic cells density, DED = dry eye disease, FL = corneal fluorescein staining score, OR = odds ratio, SjD = primary Sjögren disease.

## 4. Discussion

DED is an ophthalmic disease with multiple etiologies, of which SjD is special type. SjD affects multiple organs, and severe SjD can seriously affect a patient’s health; therefore, it is important to identify patients with SjD among individuals with DED. Previous studies have demonstrated that patients with DED and SjD exhibit more severe symptoms and have a higher corneal DCD than those without SjD.^[4]^ Yet, it remains difficult to distinguish between patients with SjD–DED and other types of DED, as well as to evaluate the activity of SjD based on DED signs.

In this study, we first analyzed differences in the signs of DED between patients with and without SjD. Previous studies have demonstrated that CND and TMH are significantly lower, while corneal DCD and corneal FL scores are significantly higher in patients with than without SjD^[5,8,9]^; however, BUT had no correlation with any of these serological markers of SjD, which is consistent with a previous study.^[9]^ Moreover, patients with DED may be treated with artificial tears, which may explain why the tear film BUT did not correlate with serological markers.

Next, we performed binary logistic regression for SjD; our results showed that CND, DCD, and TMH have good predictive values for distinguishing whether DED is caused by SjD. Patients with DED and SjD were reported to have a higher DCD,^[1,4,10,11]^ which supports our findings. Moreover, SjD can cause peripheral neuropathy, including in the subcutaneous nerves. Subcutaneous neuropathy in patients with SjD is characterized by changes in nerve density and morphology, which are believed to result from inflammation that destroys nerve fibers and interrupts nerve repair. Jamilloux et al^[12]^ found that an abnormal immunoglobulin titer was an independent predictive factor for neurological involvement in patients with SjD. One of the typical manifestations of SjD is destruction of the salivary and lacrimal glands. Our results demonstrated that TMH, which reflects the function of lacrimal secretion, is a good predictor of SjD in patients with DED.

Many previous studies have shown a correlation between autoantibody levels and clinical manifestations in patients with SjD^[13,14]^; for example, anti-SSA and anti-SSB antibodies are associated with FL of the conjunctiva and cornea.^[15]^ However, it remains difficult to evaluate the activity of the SjD based on the signs of DED. According to ESSDAI scoring, we evaluated SjD serological activity in patients with SjD–DED using IgG and C3 levels (hypocomplementemia, IgG < 5 g/L, or IgG > 16 g/L), and performed regression analyses between serological activity and DED signs. Our results demonstrate that corneal DCD and FL scores have good predictive value for evaluating the serological activity of SjD. Previous studies have shown that patients with SjD have more severe inflammation; thus, the environment, density, and morphology of dendritic cells change.^[4]^ Barcelos^[5]^ and Villani^[8]^ demonstrated that patients with SjD have a higher corneal DCD, which is associated with the degree of disease activity.^[12,16,17]^ IgG antibodies are indicators of inflammatory activity in SjD^[18]^; previous studies have shown that in patients with SjD, the levels of inflammatory factors in tears increase during inflammatory activity, which significantly correlates with corneal staining scores.^[19,20]^

Our research explored the correlation between SjD and the signs of DED, and established a binary logistic regression analysis model to predict SjD and its serological activity using the signs of DED. Corneal confocal microscopy is a commonly used examination method for patients with dry eyes in clinical practice, with the advantage of a fast, accurate, and noninvasive histological examination of the cornea. Corneal confocal microscopy can quickly obtain corneal nerve and inflammatory cell data, and combined with other indicators – such as TMH and FL – can quickly identify patients with dry eyes and SjD, as well as preliminarily evaluate their disease activity.

However, this study has the following limitations. Firstly, the sample size is relatively small, and larger datasets are needed to further validate the findings. Secondly, IVCM can provide more corneal-related data, but we only used CND and DCD, which did not fully reflect the extent of corneal damage. Furthermore, this study primarily analyzed the corneal nerves and inflammatory cells in the central corneal region, without examining the peripheral cornea. Besides, we only analyzed the serological indicators of patients with SjD, which is insufficient for a comprehensive evaluation of SjD activity.

Still, our results provide new insight. In our future research, we aim to collaborate with rheumatologists and immunologists to collect more comprehensive systemic assessment data and eye data of patients with SjD to further improve predictive models for SjD disease activity.

## 5. Conclusions

CND and TMH were found to be significantly lower, while DCD and FL scores were significantly higher in patients with than without SjD. CND, DCD, and TMH demonstrated good predictive values for distinguishing whether DED was caused by SjD, and DCD and FL scores demonstrated good predictive values for evaluating the serological activity of SjD.

## Author contributions

**Conceptualization:** Yiren Wang, Xiaodan Jiang.

**Data curation:** Yiren Wang, Jiaxi Li.

**Formal analysis:** Yiren Wang.

**Funding acquisition:** Xuemin Li.

**Investigation:** Jiaxi Li.

**Supervision:** Xiaodan Jiang, Xuemin Li.

**Writing – original draft:** Yiren Wang.

**Writing – review & editing:** Yiren Wang, Xiaodan Jiang, Xuemin Li.
